# Results of a multi-country exploratory survey of approaches and methods for IMCI case management training

**DOI:** 10.1186/1478-4505-7-18

**Published:** 2009-07-17

**Authors:** Ameena E Goga, Lulu M Muhe, Kevin Forsyth, Mickey Chopra, Samira Aboubaker, Jose Martines, Elizabeth M Mason

**Affiliations:** 1 Medical Research Council, Francie van Zijl Drive, Parowvallei, Cape Town, South Africa; 2 Department of Paediatrics and Child Health, University of Limpopo, MEDUNSA campus PO Box 197, Medunsa, 0204, South Africa; 3 Department of Child and Adolescent Health and Development (CAH), World Health Organisation, 1211 Geneva, Switzerland; 4 Flinders University, 145 Macquarie Street, Sydney 2000, Australia; 5 School of Public Health, University of the Western Modderdam Road, Bellville, Cape Town, 7535, South Africa

## Abstract

**Background:**

The Integrated Management of Childhood Illness Strategy (IMCI) is effective in improving management of sick children, and thus child survival. It is currently recommended that in-service IMCI case management training (ICMT) occur over 11-days; that the participant: facilitator ratio should be ≤4:1 and that at least 30% of ICMT time be spent on clinical practice. In 2006–2007, approximately ten years after IMCI implementation, we conducted a multi-country exploratory questionnaire survey to document country experiences with ICMT, and to determine the acceptability of shortening duration of ICMT.

**Methods:**

Questionnaires (QA) were sent to national IMCI focal persons in 27 purposively-selected countries. To probe further, questionnaires (QB and QC respectively) were also sent to course-directors or facilitators and IMCI trainees, selected using snowball sampling after applying pre-defined criteria, in these countries. Questionnaires gathered quantitative and qualitative data.

**Results:**

Thirty-three QA, 163 QB, 272 QC and two summaries were returned from 24 countries. All countries continued to adapt course content to local disease burden. All countries offer shorter ICMT courses, ranging from 3–10 days (commonest being 5–8 days). The shorter ICMT courses offer fewer exercises, more homework, less individual feedback and reduced clinical practice (<30% time). Whereas changes to course content were usually evidence-based, changes to training methodology and course duration evolved as pressure to expand implementation mounted. Participants varied in their self-reported skill and perception about each course. However, the varied methodology and integrated approach to management of illnesses were commonly cited as strengths of ICMT, and the chart booklet and clinical practice sessions were identified as critical components of ICMT. Four themes emerged from the qualitative work, viz. the current 11-day course is too expensive and should be shortened; advocacy around IMCI should increase; content should be regularly updated, new content areas should be introduced cautiously and more attention should be paid to skills-building rather than knowledge accumulation.

**Conclusion:**

Whilst the 11-day ICMT course is still recommended, as efforts intensify to increase access to quality care and meet MDG4, standardized shorter ICMT courses, that include participatory methodologies and adequate clinical practice, could be acceptable globally.

## Background

The Integrated Management of Childhood Illness Strategy (IMCI), developed by the World Health Organisation (WHO) and UNICEF in 1996, is advocated globally (and especially in developing countries) to improve the health of children and reduce under-five mortality [1]. IMCI is now implemented in over 113 countries and 5000 districts (>38% districts) across all six WHO regions [2]. IMCI includes interventions that improve health worker performance, strengthen the health system and improve household and community-based health practices, such as exclusive breastfeeding and utilisation of health services, bed nets and oral rehydration therapy [3-5]. Improving health workers skills through IMCI Case Management Training (ICMT) is one of the three components of IMCI – the others being improving the health system and improving family and community practices [6]. Eleven days is the recommended duration of training for staff at primary health care level.

To improve healthcare quality at outpatient health facilities, IMCI uses evidence-based guidelines [7] for managing the leading causes of child deaths (pneumonia, diarrhea, malaria, malnutrition, and measles) [8]. In 1996, when IMCI was launched, WHO recommended implementing the guidelines with an 11-day in-service training course for health workers and a follow-up visit to health workers' facilities one month later to reinforce new practices. Job aides (a chart booklet, a wall chart of clinical algorithms, and a one-page form for recording patient assessments, disease classifications, and treatments) were introduced to assist implementation. Specific guidelines were laid down to standardize and maintain quality of training: these stated that each course should train <24 participants; the participant:facilitator ratio should be ≤4 to 1; all training modules should be completed during a training course; each trainee should keep the IMCI chart booklet as a reference post-training; a minimum of 30% time should be spent on clinical practice and at least 20 sick children should be managed by each trainee during a training course [9]. Based on early field experience, the 1999 IMCI information package supported continued adherence to these guidelines [10]. However since 2001 global and country IMCI focal persons increasingly reported that course duration and methodology were forced to evolve as a result of limited budgets, high staff turnover and limited availability of facilitators. Furthermore coverage with and thus public access to IMCI-trained health workers remains limited – one report estimates that less than 10% of people needing IMCI-care have access to it – raising issues of child health care and health equity. Thus in an attempt to bridge the gap between policy and implementation, WHO led an exploratory survey in purposively-selected countries to gather descriptive information on four IMCI-related areas viz. (i) nationally accepted adaptations made to the training approaches, content and methods used for ICMT; (ii) facilitators' and IMCI trainees' experiences during ICMT courses (iii) IMCI trainees' perceived competence following training (iv) national focal persons', facilitators', course directors' and trainees' attitudes towards ICMT and (v) barriers to follow-up and supervision following ICMT. This paper presents the results of the first four areas of work, and is mainly descriptive, rather than analytical, in nature. To our knowledge reviews of global public health training interventions, which feed back to the source of the intervention, are limited in number, if in existence at all.

## Methods

### Study design, definitions and tools

A multi-country exploratory cross-sectional questionnaire survey of in-service ICMT approaches and methods was conducted.

We defined training approaches in terms of course duration and materials e.g. 11-continuous day course. We defined training methods as lectures, videos, role plays, written exercises and drills or other ways used to convey knowledge/skills.

To deepen our understanding we sought to gather data from key informants at various levels of the health care system – national, provincial (or state), district and sub-district levels – in each country. Three self administered questionnaires were used to gather data: Questionnaire A (QA) gathered data at national level. It comprised 36 questions about nationally-recognised ICMT courses offered. Questionnaires B (QB) and C (QC) supplemented the data obtained from Questionnaire A. QB gathered data from course facilitators/directors and comprised 17 questions in total; respondents were asked about the proportion of time spent on each content area (during courses usually facilitated or directed) and their views on the extent to which each course met its objective (scored out of 10). QC gathered data from ICMT course participants (IMCI trainees) at district or sub-district level. It comprised 23 questions (in total) that mainly focused on the trainee's experience and opinion of the course they attended and their self-reported skill post training (scored out of 10). All questionnaires gathered quantitative data, using tick boxes and closed-ended questions, and qualitative data using open-ended questions and projective techniques.

### Countries

All six WHO regions were included in the sampling frame. The 133 countries with an under-five mortality ranking lower than the median under-five mortality ranking (rank 96 or lower, or under-five mortality rate 30 more according to the State of the World's Children, UNICEF, 2005) were identified and 27 countries (including one sub-national region – Kosovo) were purposively selected from this list. Country selection criteria included a high under-five mortality rate and presence of a WHO National Programme Officer for IMCI (NPO).

### Study population

Key informants (national focal person for IMCI, National Programme Officers – NPOs -, IMCI course directors or facilitators and IMCI trainees) were purposively selected, guided by standardized criteria, using snowball sampling as the sample selection process moved from national (focal persons/NPOs) to district level (IMCI trainees). The standardized criteria specified that each country must include at least one respondent at national level (National IMCI focal person or WHO NPO); at least one IMCI course director or facilitator for each type of ICMT course being offered (they should have ever directed/facilitated two or more of this type of ICMT course) and at least one IMCI trained health worker for each type of course being offered (each trainee should have received in service training in IMCI in the past two years). We assumed that each country would be implementing two different types of IMCI courses. Based on these criteria we expected to receive 27 QA, 54 QB and 54 QC.

### Study procedures

Data were collected over 5-months (January-May 2007). Questionnaire distribution occurred via e-mail from the WHO Geneva office to WHO regional and country offices. Within each country QB and QC were distributed via e-mail or IMCI networks, depending on the availability and coverage of such networks. All respondents were informed that the data will guide WHO in its search to improve IMCI training delivery; thus they were urged to answer honestly. Respondents were only identified by a unique identifier on each form, based on standardised guidelines.

### Data analysis

Data were entered using EpiData v3.1. Data from QA, QB and QC were entered separately and cleaned (by AEG). Data from QA were analyzed using EpiData Analysis v1.1 (Build 68). In addition each QA was scrutinised for important details about the nationally recognised courses offered. Data from QB and QC were imported into SAS version 9.1 (SAS Institute Inc., Cary NC, USA) for data management and analysis. We describe and summarise the overall characteristics of the training courses currently offered, following national adaptation, in each country. As this work was set up as an exploratory survey, detailed statistical analysis was not conducted. However we did examine the perceptions and experiences of physicians (medical doctors) versus non-physicians (health officers, nurses, midwives, village health workers) to see if further hypotheses could be generated, or to check whether they any differences were statistically significant, despite the small sample. We compared the experiences of physicians versus non-physicians for shorter ICMT course (5–8 day courses) and the experiences of physicians versus non-physicians for the 11-day ICMT. We also compared physician's experiences of shorter courses with physician's experiences of the 11-day course, and non-physician's experiences of shorter courses with non-physician's experiences of the 11-day course. No further statistical analysis could be justified in view of the methods used in the study.

Qualitative data were analyzed using thematic synthesis (AEG). Qualitative data from all QA were analysed. Finally, qualitative and quantitative data were synthesized and triangulated.

## Results

QA was received from 22 countries (response rate 81.5%) (Additional file 1). QA respondents were either Ministry of Health IMCI or Child Health focal persons or WHO in-country NPOs. QB was received from 22 countries and 163 respondents (Additional file 1). Of these 114 (71%) usually directed or facilitated the recommended 11-day ICMT course whilst 47 (29%) facilitated or directed ICMT courses of varying durations (3–11 days). QC were received from 22 countries and 272 respondents (Additional file 1). Of these respondents 70% were trained after 2004, 40.5% were physicians, 30.9% were nurses, 20.2% clinical or health officers or assistants and 8.4% village health workers. QC respondents had been implementing IMCI for a median of 14 months (Q1-Q3 6–25 mo); 192 QC respondents had been trained in the 11-day ICMT course whilst 63 had received 3–14 day training.

### Adaptations to content

All 22 countries reported making several adaptations to ICMT course content (Additional file 2), following consultation with their National IMCI Adaptation Subcommittees and based on local burden of disease.

### Adaptations to course duration

All 24 countries (22 that submitted QA and 2 that submitted summaries) reported that their National Ministries of Health were officially conducting shortened ICMT courses (less than 11-days), with or without the standardized 11-day course (Additional file 3). Five countries (China, Ethiopia, Madagascar, Papua New Guinea, Peru and Sudan) were only offering shortened courses. Four countries (Eritrea, Moldova, Nigeria and Uganda) were also offering ICMT courses longer than 11-days for cadres of health workers with limited literacy.

### Adaptations to training materials and methodology

National IMCI Adaptation subcommittees had made several adaptations to training materials; these most commonly included deleting exercises, adapting drills and adding role plays (Additional file 4). The commonest changes to training methodology included reducing the number of exercises, increasing at-home reading or homework, increasing group work and reducing individual feedback.

### Adaptations to clinical practice

Countries reported having to adapt clinical practice sessions due to limited numbers of sick patients with observable clinical signs, or unavailability of transport to clinical settings. Changes included using cases in textbooks (China), increasing the number of demonstrations (Ethiopia), increasing explanation and examples before seeing patients (Cambodia), visiting sick children in acute wards on admission (Kazakhstan), seeing only outpatients on some days and only inpatients on others (Uganda), making participants switch between cases (Eritrea, Kazakhstan, Nicaragua), working in pairs (Ghana) or seeing children older than 5 years (Ethiopia).

### Detailed description of common courses

Additional file 5 presents detailed information about the common courses offered and the percentage of time spent on each content area for each of the common courses offered. Training materials for the same course duration differed between countries. Only six countries (China, Kazakhstan, Madagascar, Moldova, Sudan and Uzbekistan) reported the development of one integrated module for their shortened courses. Additional file 6 lists participants opinions of the training methods used for each ICMT course. In general 11-day ICMT participants enjoyed the integrated management, clinical practice, video, individual feedback and participatory methods but criticized the 11-day ICMT course for its recommended methodology of reading through all the modules which resulted in the course being laborious but very hurried as there was insufficient time for reading. Of QC respondents 121 (75.2%) non-physicians and 73 (71.6%) physicians were trained in 11 day courses. For the 11-day course non-physicians enjoyed the video significantly more, and tended to enjoy the at-home reading more than physicians (p = 0.005 and p = 0.007, respectively). Significantly more non-physicians than physicians thought the 11-day course was too short (p < 0.001), too hurried (p = 0.001), had too much repetition (p = 0.06) and was too laborious (p = 0.001).

Of the 40 non-physicians trained in courses other than the 11 day course, 25 (62.5%) were trained using a 5-day ICMT and 7 (17.5%) were trained in courses longer than 11 days. Of the 29 physicians trained in other courses 2 (6.9%) were trained in a 5-day course, 4 (13.8%) in a 6-day course, 3 (10.3%) in an 8-day course and 18 (62%) in courses that were 12–28 days.

With regards to shorter courses (5–8 day courses), there was a tendency for non-physicians to enjoy the video more than physicians (p = 0.09) and for physicians to identify inadequate information on HIV as a problem, more than non-physicians (p = 0.07). When we looked at physicians only, there were no significant differences in the way they experienced the shorter courses (5–8 days) versus the 11-day ICMT course, and no significant difference in how they rated their competency post-training. However when we looked at non-physicians only and compared those trained in the 11-day ICMT with those trained in shorter courses (5–8 days) the former reported significantly more enjoyment of individual feedback received during the course (p = 0.01), while the latter enjoyed the video and the integrated management more (p = 0.02 and 0.06 respectively), and complained significantly about the lack of HIV in their curricular (p < 0.001). There was a tendency for non-physician shorter course trainees to rate themselves as less skilled post-training (score 8/10, range 1–10) compared with non-physician 11-day trainees (score 8/10 range 5–10) p = 0.09.

### Results of qualitative analyses

Four themes around IMCI in general emerged from an analysis of the qualitative data (Additional file 7): the current 11-day course is too expensive; advocacy around IMCI should be increased to increase its priority; introduce new content areas cautiously, but update course content regularly; pay more attention to skills building rather than knowledge accumulation. Additional file 8 summarises respondent's views specifically on ICMT and the materials used for training.

Overall, almost all QA respondents suggested that the IMCI training approach is costly and should be revised. All respondents were of the opinion that the integrated approach to managing children is beneficial and should continue; all respondents viewed the IMCI chart booklet as an essential component of IMCI and most also saw clinical practice as an important and non-negotiable component of any ICMT course.

## Discussion

Our survey found that all countries surveyed were implementing shortened ICMT, and six were not offering the recommended 11-day course. Non-physicians seemed to benefit more from 11-day training than from training using shorter ICMT courses. Our survey is novel as it aims to systematically document experiences with training implemented as part of a global health strategy. When IMCI was conceptualized it was based on a needs assessment which quantified the extent and cause of child mortality [6] and acknowledged that vertical approaches e.g. ARI or CDD programmes have limited effectiveness as children usually suffer from more than one condition, making one single diagnosis often inadequate. Thus IMCI was developed as an integrated strategy that aims to treat major childhood illnesses and emphasises disease prevention. The training methods and standardised materials used during ICMT courses (chart booklet, wall charts, videos, photograph booklet, mother's counseling card, recording sheets and the modules) aimed to facilitated high quality classroom training and hands-on clinical practice to teach health workers how to manage sick children comprehensively [10]. Moreover tools for monitoring the process and quality of each ICMT course were also developed and made available for country use. At the time of the survey we knew that the content adaptations to IMCI had occurred [11] and that the 11-day ICMT course improves health worker performance [4,12-15].

Our survey shows that countries still conduct evidence-based adaptations to course content, regardless of course duration, based on local disease burden; for example, almost all countries with more than 5% HIV prevalence [16] have included HIV in their adaptations.

Furthermore, we found that key informants acknowledged that ICMT can potentially impact on the fourth millennium development goal (MDG4), but also stated that this impact would be maximized if training can be scaled up using shorter ICMT courses. Whereas changes to course content were usually evidence-based, changes to training methodology and course duration seemed to be organic and evolved as pressure to expand IMCI implementation mounted. Thus, except for shorter courses in Zambia and Kosovo, most other shortened courses had not been formally evaluated. A randomized trial in Zambia compared performance of primary health workers trained in the 11-day course with those trained in the six-day abridged course and found no significant difference in 10 of 12 priority and 14 of 15 supplemental indicators assessing health worker performance [17] Research in Kosovo found that assessment was either performed equally well or better by primary health care physicians trained in the 8-day course compared with those trained in the 11-day course [18]. In our survey we found that physicians seemed to manage well on both the 11-day and shorter ICMT courses. However non-physicians reportedly gained maximum benefit from the individual feedback received during 11-day training, and non-physician shorter course trainees tended to rate themselves as less skilled post-training compared with non-physician 11-day course trainees, suggesting that non-physicians needed more time for training or less course content.

A WHO consultative meeting held in Geneva in 2007, which took cognisance of this survey and other studies, recommended that a shortened course was an option for countries, providing that the course was based on core competencies (which at the time of this publication still need to be agreed upon), and complemented with opportunities for skills reinforcement and mentoring [19].

We acknowledge the limitations of this survey, including the fact that it was funded by WHO, who is also responsible for global ICMT; that countries and respondents were purposively selected; that e-mail was used to distribute some questionnaires; that sample size was not calculated so that stratified analyses by country and within country could be conducted, that the survey was cross-sectional – thus the groups compared (e.g. physicians trained in the 11-day ICMT versus physicians trained in shorter courses) were not necessarily similar, and that sample size was not calculated to measure differences between health worker performance and perspective by course duration. However our intention at this stage was to describe practices in sites already implementing IMCI, to generate hypotheses and inform further work and to provide insight into the acceptability of shorter courses. The inclusion of countries that bear the burden of under-five mortality, selection of respondents from all levels of the health system within selected countries, and the triangulation of qualitative and quantitative methods, enrich the data and add weight to the quality of information; thus the survey provides useful guidance at a global level as ways to improve delivery of ICMT are sought.

The data from this survey emphasises the need for constant review of the approaches used to increase health worker skills at a programmatic level. Although most public health approaches are difficult to evaluate due to lack of clearly-stated objectives or criteria for training [20] and the short time scales from initiation to assessment [21], a global review of ICMT is simplified by several factors including the availability of clear implementation guidelines for ICMT and the fact that most countries have been running ICMT courses for 5–10 years. Thus regular reviews of ICMT are possible and would place IMCI in a better position to effectively contribute towards meeting MDG4.

## Conclusion

This survey showed that selected countries invariably made adaptations to ICMT courses based on country needs. Countries have also developed shortened ICMT courses, mostly in addition to the standard 11-day course. Taking cognizance of these findings, of the demand for shortened ICMT courses, of the suggestions made by survey respondents and of the discussions at the 2007 WHO Technical Consultation [19] we conclude that there is a place for a standardized shortened course to increase options for ICMT. However we believe that a shortened course should be geared towards appropriately selected target audiences, based on core competencies and supplemented with follow up and mentoring post-training.

## Abbreviations

This paper uses the following abbreviations: ARI: Acute Respiratory Infection; CDD: Control of Diarrhoeal Disease; IMCI: Integrated Management of Childhood Illness Strategy; ICMT: IMCI Case Management Training; MDG: Millennium Development Goals; NPO: WHO National Programme Officer for IMCI; QA: Questionnaire A; QB: Questionnaire B; QC: Questionnaire C; UNICEF: United Nations Children's Fund; WHO: World Health Organisation.

## Competing interests

This work was undertaken as part of WHO-funded monitoring and evaluation of a public health strategy being implemented globally by Ministries of Health. The work was funded by the World Health Organisation, who, together with UNICEF, also developed the IMCI strategy.

AEG was employed as a WHO consultant during the time of this work.

LM, SA, JM and EMM work at the Work Health Organisation, Geneva.

However all authors had no personal interest in the results of this survey, and have represented the results as they were gathered.

## Authors' contributions

AEG conceptualized the study, developed the tools, conducted the data analysis and wrote the first and final drafts of the manuscript. LM assisted with conceptualization, tool development and manuscript development and finalization. EM, SA, JM assisted with conceptualisation, tool development and manuscript development and finalization

MC assisted with manuscript development and finalization. All authors read and approved the final manuscript.

## Supplementary Material

Additional file 1 **Table 1: Questionnaires received from regions and countries**. This table describes the number of questionnaires received from regions and countries Click here for file

Additional file 2 **Table 2: Adaptations made to the content of IMCI case management training, by country**. This table summarises the adaptations made to the content of IMCI case management training Click here for file

Additional file 3 **Table 3: Adaptations made to the duration of IMCI case management training, and countries making these adaptations**. This table summarises the adaptations made to the duration of IMCI Case management training Click here for file

Additional file 4 **Table 4: Adaptations made to IMCI training materials, by country**. This table summarises the adaptations made to IMCI training materials by country Click here for file

Additional file 5 **Table 5: Median percentage of time reportedly spent on each IMCI module by 5, 6, 7–8 and 11-day courses**. This table presents the median percentage of time that 5, 6, 7–8 and 11 day courses spent on each module of IMCI Click here for file

Additional file 6 **Table 6: Participants' experiences of the course they attended**. This table presents participants experiences of the IMCI training course they attended Click here for file

Additional file 7 **Panel I: Suggestions from respondents**: This panel presents the suggestions that respondents made regarding IMCI case management training Click here for file

Additional file 8 **Panel II: Selected quotes from respondents**: This figure presents selected quotes from respondents about IMCI Case management training Click here for file
