# Supplementary material for: Results of a multi-country exploratory survey of approaches and methods for IMCI case management training
Source: Health Res Policy Syst. 2009 Jul 17;7:18. doi: 10.1186/1478-4505-7-18 (PMC2723104; doi:10.1186/1478-4505-7-18)
Supplement: Additional file 1 — Table 1: Questionnaires received from regions and countries. This table describes the number of questionnaires received from regions and countries [file 1478-4505-7-18-S1.doc]

*Table 1:* Questionnaires received from regions and countries

| **Region / Country** | **QA n=33** | **QB n=163** | **QC**  **n= 272** | **Total no. questionnaires**  **n=473** |
| --- | --- | --- | --- | --- |
| **AFRO** | **12 (36.4)** | **73 (44.8)** | **100 (36.8)** | **185 (39.1)** |
| Eritrea | 2 (6.1) | 10 (6.1) | 6 (2.2) | 18 |
| Ethiopia | 1 (3.0) | 13 (8.0) | 4 (1.5) | 18 |
| Ghana | 1 (3.0) | 9 (5.5) | 22 (8.2) | 32 |
| Kenya | 2 (6.1) | 1 (0.6) | 1 (0.4) | 4 |
| Madagascar | 3 (9.1) | 7 (4.3) | 2 (0.7) | 12 |
| Niger | 0 | 7 (4.3) | 13 (4.8) | 20 |
| Nigeria | 1 (3.0) | 4 (2.4) | 13 (4.8) | 18 |
| United Republic of Tanzania | 1 (3.0) | 15 (9.2) | 31 (11.5) | 47 |
| Uganda | 1 (3.0) | 3 (1.8) | 2 (0.7) | 6 |
| Zambia | 0 | 4 (2.45) | 6 (2.2) | 10 |
| **WPRO** | **5 (15.1)** | **54 (33.1)** | **108 (39.7)** | **167 (35.3)** |
| Cambodia | 2 (6.1) | 5 (3.1) | 16 (6.0) | 23 |
| China | 1 (3.0) | 18 (11.0) | 15 (5.6) | 34 |
| Fiji | 1 (3.0) | 2 (1.2) | 1 (0.4) | 4 |
| Papua New Guinea | 0 | 0 | 5 (1.9) | 5 |
| Vietnam | 1 (3.0) | 24 (14.7) | 71 (26.4) | 96 |
| **SEARO** | **4 (12.1)** | **3 (1.8)** | **9 (3.3)** | **16 (3.4)** |
| India | 2 (6.1) | 1 (0.6) | 8 (3.0) | 11 |
| Indonesia | 1 (3.0) | 2 (1.2) | 1 (0.4) | 4 |
| Nepal | 1 (3.0) | 0 | 0 | 1 |
| **EURO** | **9 (27.3)** | **35 (21.5)** | **52 (19.1)** | **96 (20.3)** |
| Kazakhstan | 2 (6.1) | 18 (11.0) | 8 (3.0) | 28 |
| Kosovo | 2 (6.1) | 3 (1.8) | 2 (0.7) | 7 |
| Republic of Moldova | 2 (6.1) | 9 (5.5) | 13 (4.8) | 24 |
| Uzbekistan | 3 (9.1) | 5 (3.1) | 29 (9.7) | 37 |
| **EMRO** | **1 (3.0)** | **0** | **0** | **1 (0.2)** |
| Sudan | 1 (3.0) | 0 | 0 | 1 |
| Jordan* | 0 | 0 | 0 |  |
| Egypt* | 0 | 0 | 0 |  |
| **AMRO/PAHO** | **2 (6.1)** | **3 (1.8)** | **3 (1.1)** | **8 (1.7)** |
| Peru | 1 (3.1) | 2 (1.2) | 0 | 3 |
| Nicaragua | 1 (3.1) | 1 (0.6) | 3 (1.2) | 5 |

*Footnote:* * The Eastern Mediterranean Regional office of WHO sent a summary document on experiences and adaptations in Egypt and Jordan.
