# Supplementary material for: Results of a multi-country exploratory survey of approaches and methods for IMCI case management training
Source: Health Res Policy Syst. 2009 Jul 17;7:18. doi: 10.1186/1478-4505-7-18 (PMC2723104; doi:10.1186/1478-4505-7-18)
Supplement: Additional file 2 — Table 2: Adaptations made to the content of IMCI case management training, by country. This table summarises the adaptations made to the content of IMCI case management training [file 1478-4505-7-18-S2.doc]

*Table 1:* Adaptations made to the content of IMCI case management training, by country

| **REGION** | **COUNTRY** | **Care of the Newborn** | **Wheeze** | **Sore Throat** | **Fever** | **Dengue** | **HIV diagnosis** | **HIV classification** | **ARVs** | **HIV & IF** | **Malaria diagnosis** | **Anaemia diagnosis** | **Skin** | **Accidents** | **Care for devtelopment** |
| --- | --- | --- | --- | --- | --- | --- | --- | --- | --- | --- | --- | --- | --- | --- | --- |
| AFRO | Eritrea | √ |  |  | √ |  |  |  |  |  |  |  |  |  |  |
|  | Ethiopia | √ |  |  | √ |  | √ | √ |  | √ |  |  |  |  |  |
|  | Ghana |  |  |  | √ |  |  |  |  |  |  |  |  |  |  |
|  | Kenya^a^ | √ | √ |  | √ |  | √ | √ |  | √ |  |  |  |  | √ |
|  | Madagascar | √ |  |  | √ |  |  |  |  |  | √ |  |  |  |  |
|  | Niger | √ |  |  | √ |  |  |  |  |  | √ | √ |  |  |  |
|  | Nigeria | √ |  |  | √ |  |  |  | √ |  |  |  |  |  |  |
|  | United Rep. of Tanzania | √ | √ |  |  |  | √ | √ |  |  |  |  |  |  |  |
|  | Uganda | √ |  |  |  |  | √ | √ |  |  |  |  |  |  |  |
|  | Zambia |  |  |  | √ |  |  |  |  |  |  |  |  |  |  |
| WPRO | Cambodia | √ |  |  | √ | √ |  |  |  |  | √ |  | √ |  |  |
|  | China |  |  |  | √ | √ |  |  |  |  | √ |  |  |  |  |
|  | Fiji |  |  |  | √ |  |  |  |  |  | √ |  |  |  |  |
|  | Papua New Guinea |  |  |  | √ |  |  |  |  |  |  |  |  |  |  |
|  | Vietnam |  |  |  |  |  |  |  |  |  |  |  |  |  | √ |
| SEARO | India | √ |  |  | √ |  | √ | √ | √ |  | √ |  |  |  |  |
|  | Indonesia | √ |  |  | √ | √ |  |  |  |  |  |  |  |  |  |
|  | Nepal |  | √ |  | √ |  |  | √ | √ | √ | √ |  |  |  |  |
| EURO | Kazakhstan |  | √ | √ | √ | √ |  |  |  |  |  |  |  | √ | √ |
|  | Kosovo | √ | √ | √ |  |  |  |  |  |  |  |  |  |  |  |
|  | Republic of Moldova |  | √ | √ | √ |  |  |  |  |  |  |  |  |  | √ |
|  | Uzbekistan |  | √ | √ | √ |  |  |  |  |  |  |  |  | √ | √ |
| EMRO | Sudan | √ | √ |  | √ |  |  |  |  |  |  |  |  |  |  |
| PAHO | Peru | √ | √ | √ | √ | √ | √ | √ |  |  |  | √ | √ | √ |  |
|  | Nicaragua |  |  |  | **√** |  |  |  |  |  |  |  |  |  |  |
