# Supplementary material for: Results of a multi-country exploratory survey of approaches and methods for IMCI case management training
Source: Health Res Policy Syst. 2009 Jul 17;7:18. doi: 10.1186/1478-4505-7-18 (PMC2723104; doi:10.1186/1478-4505-7-18)
Supplement: Additional file 3 — Table 3: Adaptations made to the duration of IMCI case management training, and countries making these adaptations. This table summarises the adaptations made to the duration of IMCI Case management training [file 1478-4505-7-18-S3.doc]

*Table 1:* Countries that have made adaptations made to the duration of IMCI case management training

| **Course duration** | | | | | | | | | | |
| --- | --- | --- | --- | --- | --- | --- | --- | --- | --- | --- |
| **3-day** | **4-day** | **5-day** | **6-day** | **7-day** | **8-day** | **10-day** | **11-day** | **12-day** | **13-day** | **14-day** |
| Ghana  Indonesia  Nicaragua  Madagascar | Kenya  Uzbekistan (course on community IMCI) | China  Fiji  Indonesia  Kazakhstan  Nicaragua  Nigeria  Madagascar  Peru  Sudan | Ethiopía  India  Indonesia  Kazakhstan  Madagascar  Nigeria  Niger  Uganda | China  Egypt  Jordan  Nepal  Peru | Egypt  India  Jordan  Kosovo | Kosovo | Offered by **all** countries **except** China, Ethiopia, Madagascar, Papua New Guinea, Peru and Sudan | Moldova |  | Eritrea  Nigeria  Uganda |
