# Supplementary material for: Results of a multi-country exploratory survey of approaches and methods for IMCI case management training
Source: Health Res Policy Syst. 2009 Jul 17;7:18. doi: 10.1186/1478-4505-7-18 (PMC2723104; doi:10.1186/1478-4505-7-18)
Supplement: Additional file 4 — Table 4: Adaptations made to IMCI training materials, by country. This table summarises the adaptations made to IMCI training materials by country [file 1478-4505-7-18-S4.doc]

*Table 1:* Adaptations made to IMCI training materials by country

| **REGION** | **COUNTRY** | **Exercises added** | **Exercises reviewed and changed** | **Drills added** | **Drills adapted** | **Local video developed** | **Local photo booklet developed** | **Role plays added** | **Other** |
| --- | --- | --- | --- | --- | --- | --- | --- | --- | --- |
| AFRO | Eritrea |  |  |  |  |  |  |  |  |
|  | Ethiopia | √ | √ |  | √ |  |  |  |  |
|  | Ghana |  | √ |  |  |  |  |  |  |
|  | Kenya^a^ | √ | √ |  |  |  |  | √ |  |
|  | Madagascar | √ | √ |  | √ |  |  | √ |  |
|  | Niger |  | √ |  | √ |  |  | √ |  |
|  | Nigeria | √ | √ | √ |  |  |  | √ |  |
|  | United Rep. of Tanzania |  |  |  | √ |  |  | √ |  |
|  | Uganda |  |  |  |  |  |  |  |  |
|  | Zambia |  |  |  |  |  |  |  |  |
| WPRO | Cambodia |  |  |  |  |  |  |  | √ ^a^ |
|  | China |  | √ |  |  |  |  |  |  |
|  | Fiji | √ | √ | √ | √ |  |  | √ |  |
|  | Papua New Guinea |  |  |  |  |  |  |  |  |
|  | Vietnam |  | √ |  | √ |  |  |  |  |
| SEARO | India |  | √ |  | √ | √ | √ | √ | √^b^ |
|  | Indonesia |  | √ |  | √ |  | √ |  |  |
|  | Nepal |  | √ |  |  | √ |  |  | √^c^ |
| EURO | Kazakhstan | √ | √ |  | √ |  |  |  |  |
|  | Kosovo |  | √ |  | √ |  |  |  |  |
|  | Republic of Moldova | √ | √ |  | √ |  |  |  |  |
|  | Uzbekistan |  | √ |  | √ | √ |  |  |  |
| EMRO | Sudan | √ | √ | √ | √ |  |  |  |  |
| PAHO | Peru | √ | √ |  | √ |  |  | √ |  |
|  | Nicaragua |  |  |  |  |  |  |  |  |

*Footnotes*: **a**: MRDT practical sessions added; **b:** different packages for different workers **c:** few exercises removed.
