# Supplementary material for: Results of a multi-country exploratory survey of approaches and methods for IMCI case management training
Source: Health Res Policy Syst. 2009 Jul 17;7:18. doi: 10.1186/1478-4505-7-18 (PMC2723104; doi:10.1186/1478-4505-7-18)
Supplement: Additional file 5 — Table 5: Median percentage of time reportedly spent on each IMCI module by 5, 6, 7–8 and 11-day courses. This table presents the median percentage of time that 5, 6, 7–8 and 11 day courses spent on each module of IMCI [file 1478-4505-7-18-S5.doc]

*Table 1:* Median percentage of time reportedly spent on each IMCI module by 5, 6, 7-8 and 11-day courses

| **Module** | **Recommendation** | **Actual time spent** | | | |
| --- | --- | --- | --- | --- | --- |
|  | **% of course time that should be spent** | **5-day course**  **% (range)** | **6-day course**  **% (range)** | **7-8 day course**  **% (range)** | **11-day course**  **% (range)** |
| Introduction | 1 | 5 (3-5) | 2.5 (2-3) | 4 (2.5-25) | 2.45 (1.1-10) |
| Assess & Classify | 21 | 20 (17.5-30) | 12.5 (10-15) | 20 (10-35) | 20 (2.8-45) |
| Identify Treatment | 5 | 10 (5-15) | 6.5 (5-8) | 6.5 (2-10) | 7 (2-20) |
| Treat | 12 | 10 (5-25) | 9 (8-10) | 10 (0-20) | 11.1 (3-40) |
| Follow-up | 7 | 5 (5-10) | 3.5 (2-5) | 5 (5-10) | 5 (2-15) |
| Counsel | 7 | 10 (9-50) | 6 (5-7) | 10 (3-20) | 9 (2-20) |
| Sick Young Infant | 3 | 10 (10-15) | 15 (15) | 12 (1-25) | 10 (3.6-20) |
| Clinical | 44 | 20 (20-50) | 45 (40-50) | 37.5 (5-41) | 30.4 (5-50) |

*Footnote:* Data on percentage of time usually spent on each content area are from QB. Note the IMCI information package states that not less than 30% of ICMT course time should be spent on clinical practice
