# Supplementary material for: Results of a multi-country exploratory survey of approaches and methods for IMCI case management training
Source: Health Res Policy Syst. 2009 Jul 17;7:18. doi: 10.1186/1478-4505-7-18 (PMC2723104; doi:10.1186/1478-4505-7-18)
Supplement: Additional file 6 — Table 6: Participants' experiences of the course they attended. This table presents participants experiences of the IMCI training course they attended [file 1478-4505-7-18-S6.doc]

*TABLE 1:* Participants experiences of the course they attended

|  | **6-day course** | **7-8 day course** | **11-day IMCI training** | | | | |
| --- | --- | --- | --- | --- | --- | --- | --- |
|  |  |  | **Physicians**  **n=65** | **Non-physicians**  **110** | **Total** | **p-value** |  |
| Participants rating of competency following course***** | 8/10  (7-10) | 6.5/10  (4-10) | 8/10  (4-10) | 8/10  (5-10) | 8/10  (4-10) | 0.8 |  |
| Course director’s rating of course**^** | 7.7/10  (5-9) | 8/10  (7-9) | NA | NA | **^** 8/10 (1-10) |  |  |
| **No and (%) part who enjoyed the following aspects of each course** | | | | | | | |
| Integrated Management | 4 (100) | 5 (83.3) | 66 (92.9) | 107 (89.9) | 173 (91.0) | 0.6 |  |
| Clinical sessions | 4 (100) | 6 (100) | 58 (81.7) | 91 (76.5) | 149 (78.4) | 0.5 |  |
| Classroom reading | 3 (75) | 6 (100) | 36 (50.7) | 58 (48.7) | 94 (49.47) | 0.9 |  |
| At-home reading | 2 (50) | 3 (50) | 16 (22.5) | 50 (42.0) | 66 (34.74) | 0.007^╪^ |  |
| Individual feedback | 3 (75) | 1 (16.7) | 50 (7.4) | 81 (68.1) | 131 (68.95) | 0.7 |  |
| Partic. Method | 4 (100) | 4 (66.7) | 49 (69.0) | 81 (68.1) | 130 (68.4) | 1 |  |
| Exercises | 4 (100) | 5 (83.3) | 45 (63.4) | 76 (63.9) | 121 (63.7) | 1 |  |
| Drills | 3 (75) | 5 (83.3) | 45 (63.4) | 74 (62.2) | 119 (62.6) | 1 |  |
| Video | 4 (100) | 4 (66.7) | 40 (56.3) | 91 (76.5) | 131 (69.0) | 0.005^╪^ |  |
| Counselling skills | 3 (75) | 6 (100) | 50 (70.4) | 72 (60.5) | 122 (64.2) | 0.2 |  |
| Role plays | 4 (100) | 5 (83.3) | 50 (70.4) | 74 (62.2) | 124 (65.3) | 0.3 |  |
| Discussions | 3 (75) | 3 (50) | 39 (54.9) | 72 (60.5) | 111 (58.4) | 0.5 |  |
| Skills of facilitators | 3 (75) | 2 (33.3) | 44 (62) | 77 (64.7) | 121 (63.7) | 0.8 |  |
| Appropriate pace of teaching | 4 (100) | 2 (33.3) | 32 (45.1) | 53 (44.5) | 85 (44.7) | 1 |  |
| Variety of teaching methods | 3 (75) | 5 (83.3) | 51 (71.8) | 77 (64.1) | 128 (67.4) | 0.3 |  |
| **No and (%) who identified gaps in course** | | | | | | | |
| Too detailed | 0 | 0 | 7 (9.9) | 11 (9.2) | 18 (9.5) | 1 |  |
| Didactic | 0 | 0 | 3 (4.2) | 13 (17.8) | 16 (8.4) | 0.2 |  |
| Too few exercises | 1 (25) | 0 | 0 | 12 (10.7) | 12 (6.35) |  |  |
| Too short | 1 (25) | 2 (33.3) | 8 (11.3) | 58 (49.2) | 66 (34.9) | <0.001^╪^ |  |
| Too hurried / fast | 1 (25) | 5 (83.3) | 12 (16.94) | 64 (54.2) | 76 (40.2) | 0.001^╪^ |  |
| Too much repetition | 0 | 0 | 7 (9.9) | 8 (6.8) | 15 (7.9) | 0.06 |  |
| Too laborious | 0 | 1 (16.7) | 6 (8.4) | 33 (28.0) | 39 (20.6) | 0.001^╪^ |  |
| Did not focus on HIV | 1 (25) | 1 (16.7) | 21 (29.6) | 51 (43.2) | 72 (38.1) | 0.06 |  |
| Did not focus on neonates | 0 | 0 | 22 (31.0) | 25 (21.2) | 47 (24.9) | 0.2 |  |

Footnotes: *median score out of 10 (range); ^ median score out of 10 (range); For the 6-day course data on course-director rating of the course are from 14 QB sent by course directors / facilitators in Ethiopia (3), Indonesia (1), Kazakhstan (2), Madagascar (5), Nigeria (1), Peru (1) and Tanzania (1). For the 7-8 day course data on course-director rating of the course are from 2 QB sent by course directors / facilitators in Peru (1 respondent) and India (1 respondent). For the 11-day course data on course-director rating of the course are from 94 QB sent by course directors / facilitators in Cambodia (5 respondents), Eritrea (6 respondents), Ethiopia (6), Fiji (2), Ghana (8), Indonesia (1), Kazakhstan (10), Kenya (1), Kosovo (1), Madagascar (1), Niger (3), Nigeria (2), Tanzania (12), Uganda (3), Uzbekistan (5), Vietnam (24), Zambia (4). For the 6-day, 7-8 day, 11-day course respectively data on what participants enjoyed about the course and gaps / criticisms of the course are from QC, 6 QC, 190 QC. NA – data not gathered. ^╪^ p<0.05.
