# Supplementary material for: Results of a multi-country exploratory survey of approaches and methods for IMCI case management training
Source: Health Res Policy Syst. 2009 Jul 17;7:18. doi: 10.1186/1478-4505-7-18 (PMC2723104; doi:10.1186/1478-4505-7-18)
Supplement: Additional file 7 — Panel I: Suggestions from respondents: This panel presents the suggestions that respondents made regarding IMCI case management training [file 1478-4505-7-18-S7.doc]

**Panel I: Themes that emerged from an analysis of the qualitative data, and quotes that contribute towards each theme:**

**Theme 1: The current 11-day course is too expensive and should be improved by shortening course duration, simplifying materials and updating facilitators:**

- *Duration too long, requires too many trainers and large budget*
- *Shorten IMCI training course*
- *Reduce reading*
- *Course duration should be not more than 5 days. Increase the number participants and facilitators per course*
- *HQ and AFRO need to develop a shorter course - 6 days*
- *IMCI should be modular -to prevent long stay away from clinic*
- *11 day course too long and expensive, introduce homework to decrease duration*
- *Introduce 2 versions of IMCI training: 11-day and shortened 6-7 day*
- *Shorten training to 7-8 days*
- *Develop guiding model for standard on the job or distance learning*
- *Add job aids for session summaries*
- *Adopt training method of infant and young child feeding - group all participants together so facilitators can work in turns*
- *Shorten course materials - at least for doctors*
- *Can ‘WHO’ make a crash course / module for staff who cannot be available for 11 day*
- *Facilitators need updating and refreshing*
- *Emphasise module on counselling*

**Theme 2: Advocacy around IMCI should be increased so that IMCI is prioritized as a key intervention within Ministries of Health and amongst important donors**

**Theme 3: Introduce new content areas cautiously, but update course content regularly**

- *Inclusion of new topics decreases overall quality*
- *Cover most essential aspects for child survival but do not overload the course*
- *Update content every 5 years*
- *HIV should be introduced as a complementary course*
- *Simplify materials for lower level cadres*

**Theme 4: Change the focus and content of training: more attention should be paid to skills-building rather than knowledge accumulation**

- *Pay more attention to change of practice rather than knowledge and skill*
- *Non-doctors should be allowed to facilitate IMCI course. Reduce reading*
- *Need more time for clinical practice*
- *Decrease number of modules; decrease exercises, GE and ARI can be decreased*
- *Different courses, of different depth are needed by different cadres of health workers. However all courses must use the IMCI CB*
- *For implementers at primary heath care level a shorter, action-orientated course with less reading is needed*
- *Managers need shorter course with an in-depth focus on systems strengthening and how to integrate IMCI into routine district management*
- *Academics need a course that explains the rationale / evidence behind IMCI*
