# Supplementary material for: Results of a multi-country exploratory survey of approaches and methods for IMCI case management training
Source: Health Res Policy Syst. 2009 Jul 17;7:18. doi: 10.1186/1478-4505-7-18 (PMC2723104; doi:10.1186/1478-4505-7-18)
Supplement: Additional file 8 — Panel II: Selected quotes from respondents: This figure presents selected quotes from respondents about IMCI Case management training [file 1478-4505-7-18-S8.doc]

**Panel II: Respondents views on ICMT training and materials (Selected quotes from**

**respondents):**

### Respondents’ views on the IMCI chart booklet

- *Very good tool in managing child health*
- *The bible of IMCI / the bible of child survival* (almost half respondents in Forms A, B and C combined made this statement)
- *Colourful, convenient, informative, good job aids, but expensive to copy*
- *Concise clear and very useful*
- *I'm lucky to have it*
- *Concise book - has been compared to Hindu's holy book -the GEETA*
- *Easy to follow*
- *Summary of IMCI in a nutshell*
- *Useful tool but can't refer in front of patients*
- *Daily use*

**Respondent’s views on IMCI training courses in general**

- *Very good but too much to read*
- *Necessary for PHC workers*
- *I was told it was a good training but long*
- *Intensive course*
- *Need to be changed for different situations*
- *Very useful for me*
- *New way of training that has opened doors to other training*
- *Good training methodology but too costly*
- *Very useful, but difficult and demanding*
- *Hectic reading and exercises*
- *The answer to improving child survival*

**Respondents views on the 11-day IMCI training course**

- *Tiring but productive training*
- *Long, good but too long - why is it too long?*
- *Course too short for 1st level health workers, but too long for managers*
- *Expensive. Difficult for PHC facilities to find replacement personnel*
- *Long day. Good food, exercises, clinical practice*
- *Long training course, too long a working day*
- *Very exhausting but effective*
- *Perfect ideal course and duration*
- *11-days of hard, intense and committed work.. But fun*
- *Two weeks off routine health facility work*
- *Tedious*
